# Supplementary material for: STEM enables mapping of single-cell and spatial transcriptomics data with transfer learning
Source: Commun Biol. 2024 Jan 6;7:56. doi: 10.1038/s42003-023-05640-1 (PMC10771471; doi:10.1038/s42003-023-05640-1)
Supplement: Supplementary file 2 — Supplementary Information [file 42003_2023_5640_MOESM2_ESM.pdf]

Supplementary Materials of STEM enables mapping single-cell and spatial transcriptomics data with transfer learning

Supplementary Figures

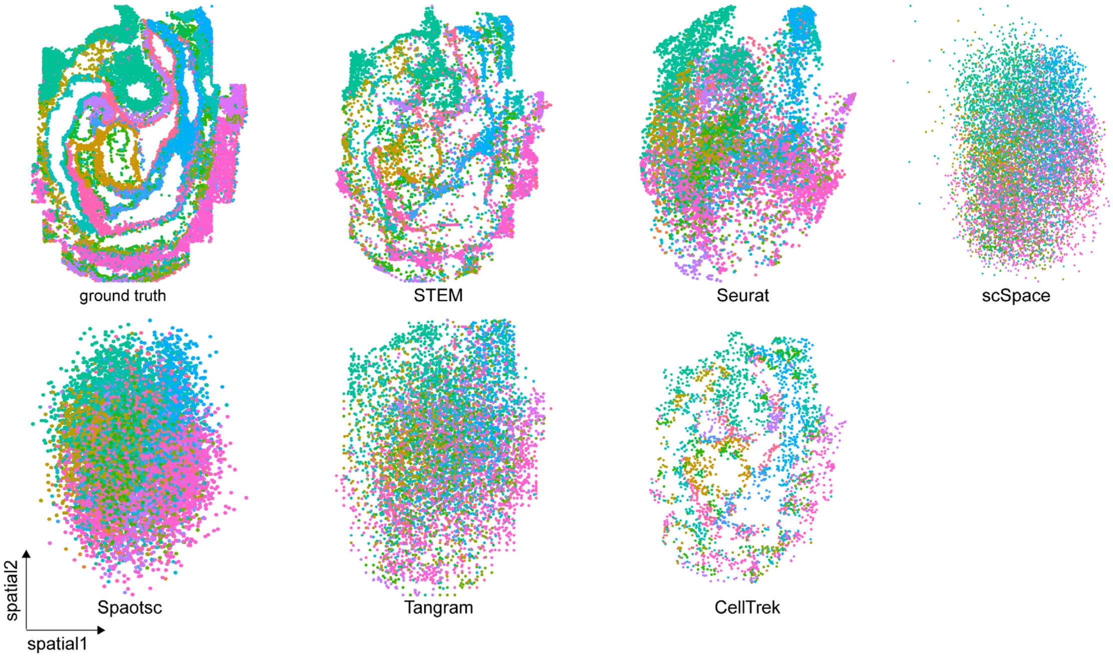

Supplementary Figure 1. The spatial reconstruction results of all methods on embryo 1.

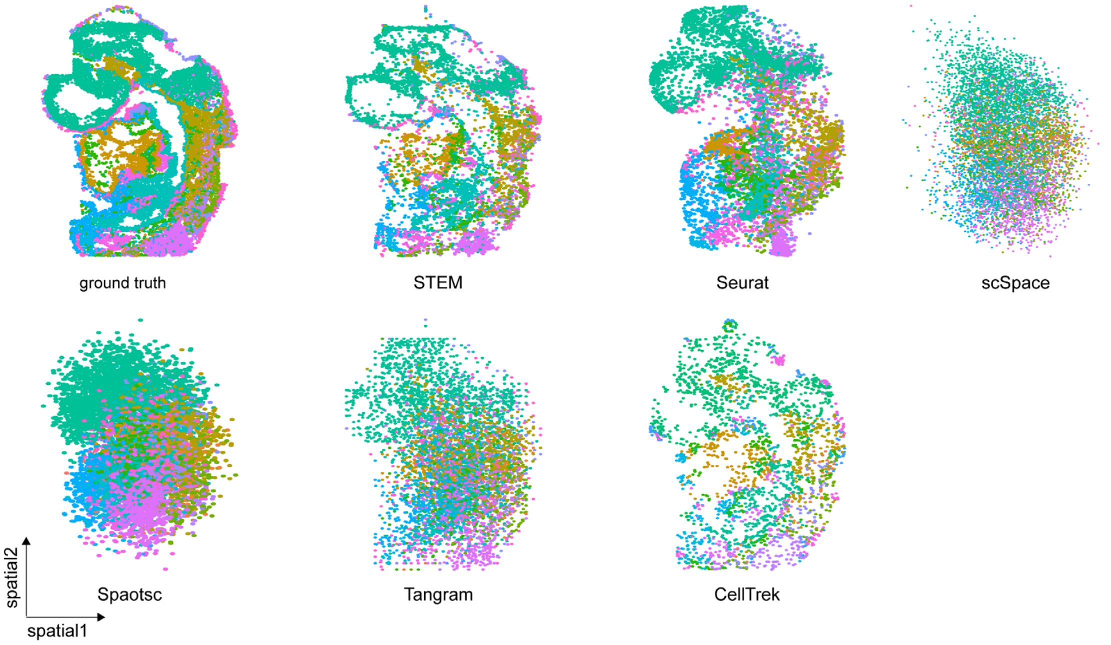

Supplementary Figure 2. The spatial reconstruction results of all methods on embryo 2.

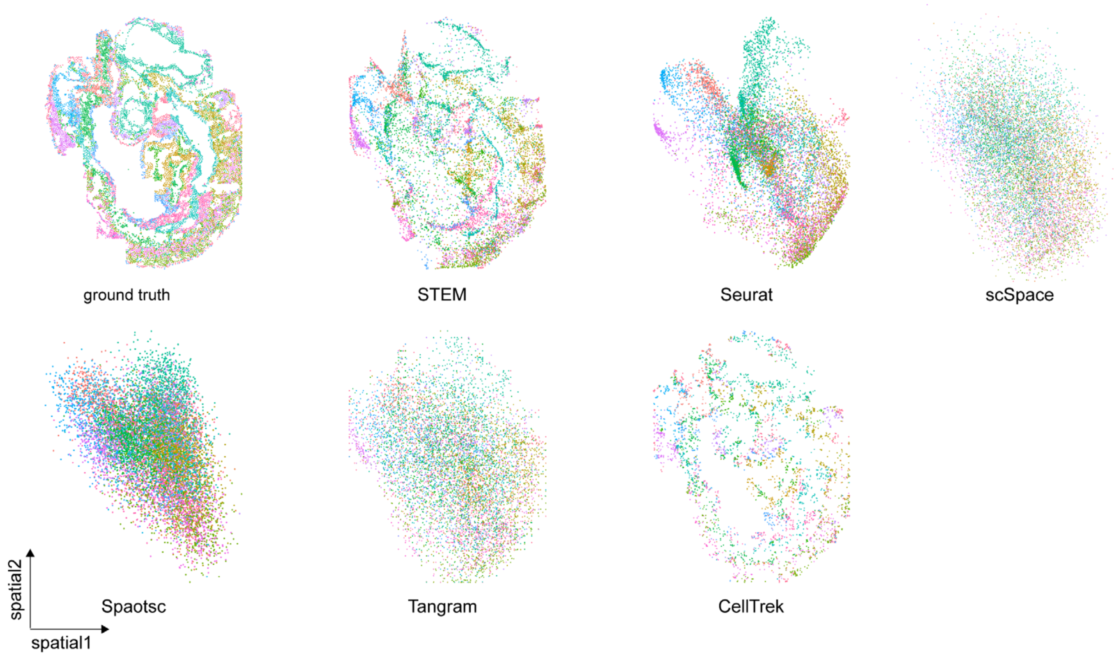

Supplementary Figure 3. The spatial reconstruction results of all methods on embryo 3.

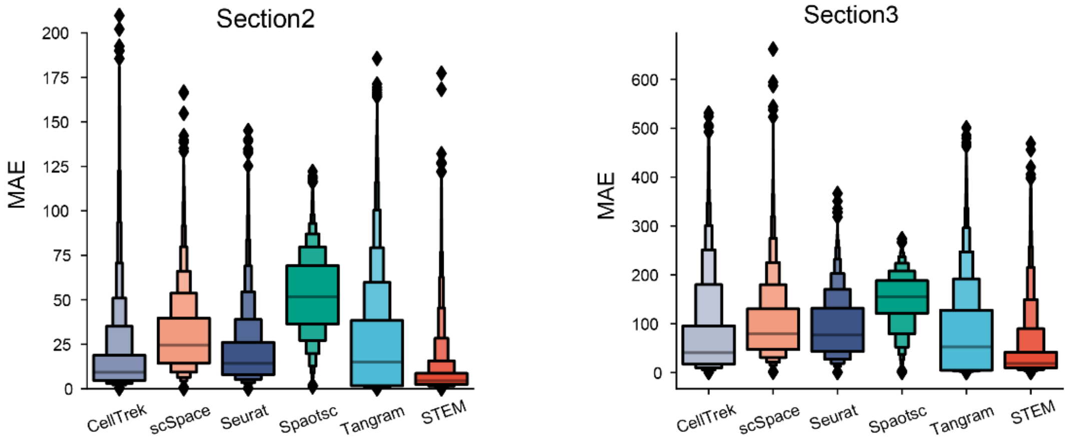

Supplementary Figure 4. The enhanced boxplot of means absolute error (MAE) computed by different methods' results on the mouse embryos 2 and 3. The horizontal bar inside the box represents the median of all values. Each edge of the box represents the half percentiles of the rest data, in other words, splitting the rest data into two halves.

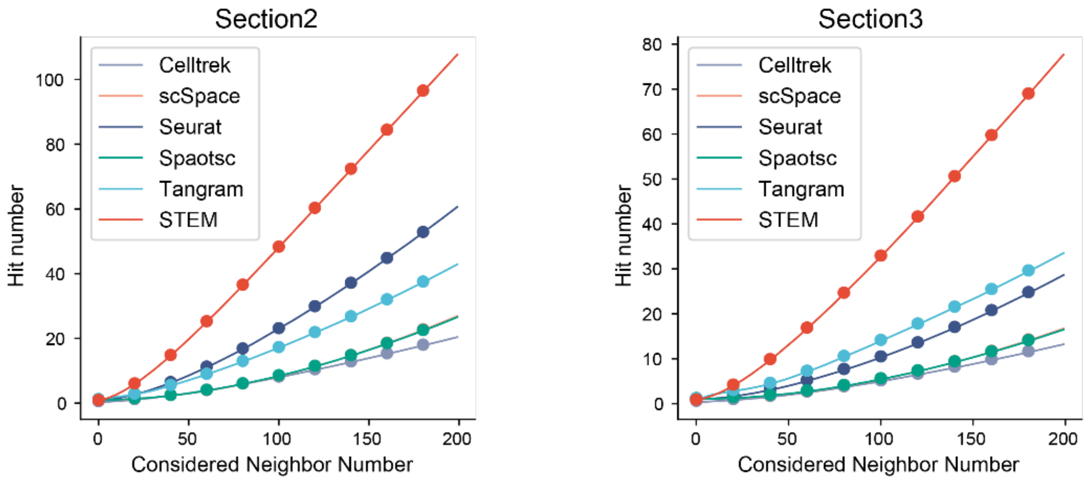

Supplementary Figure 5. The Hit number computed by different methods' results under different considered neighbor numbers on the mouse embryos 2 and 3.

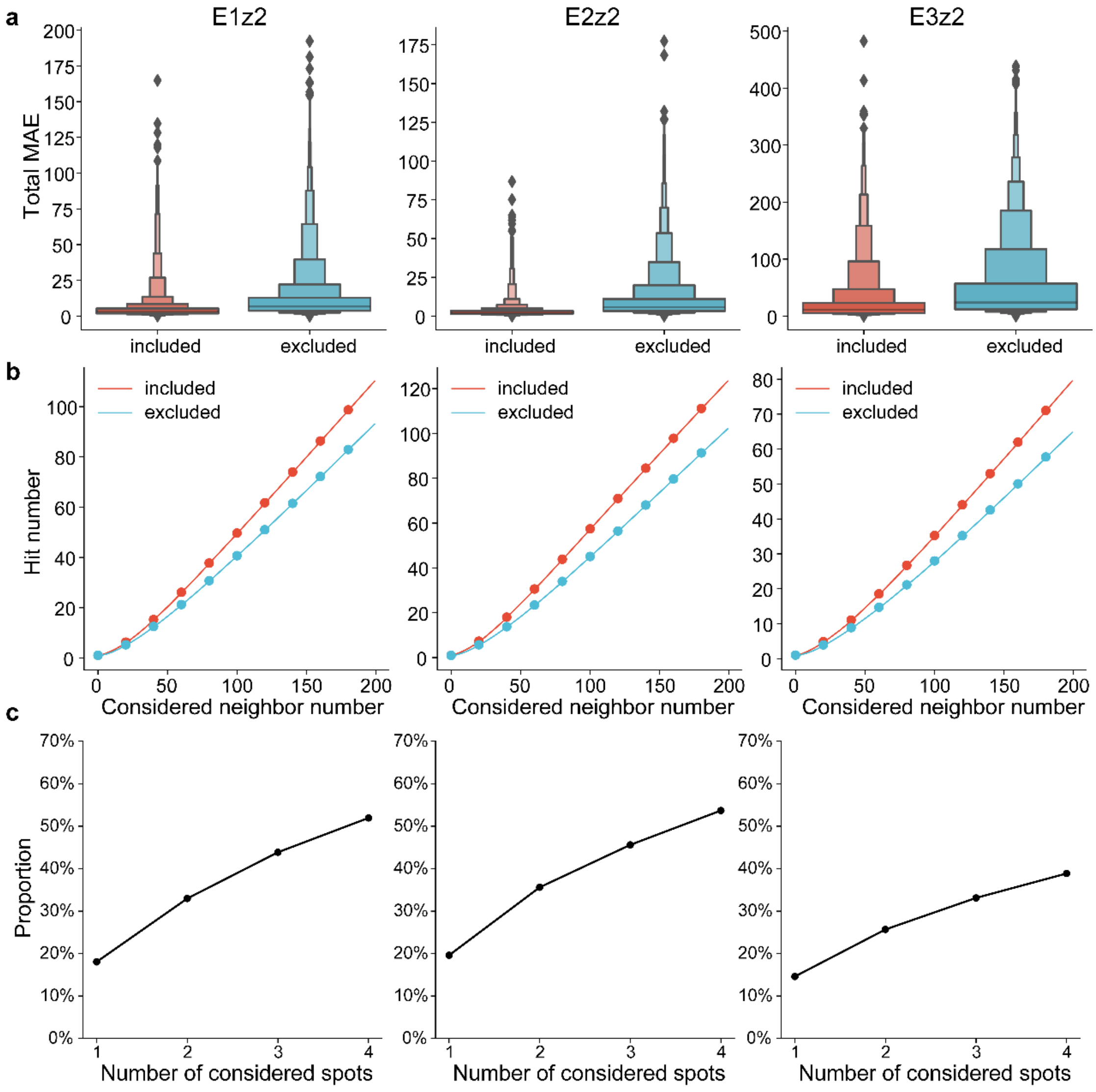

Supplementary Figure 6. The performance of "include" and "exclude" groups on semi-simulation data. The "include" and "exclude" groups contain cells that are included or

excluded in the spot. (a) The total MAE of two cell groups given by STEM. In this enhanced box plot, the horizontal bar inside the box represents the median of all values. Each edge of the box represents the half percentiles of the rest data, in other words, splitting the rest data into two halves. (b) The hit number performance of two cell groups given by STEM. (c) The proportion of excluded cells that were mapped into the K closest spots. The x-axis is the K values, and Y axis is the proportion.

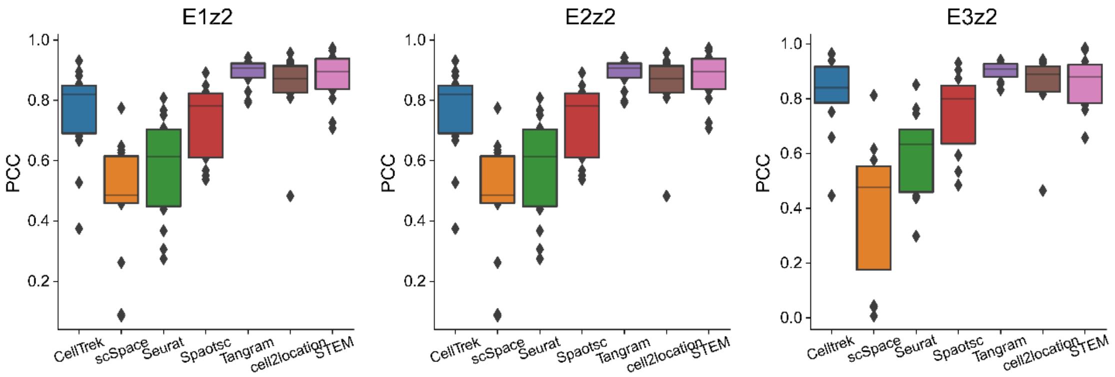

Supplementary Figure 7. The Pearson correlation coefficient (PCC) was computed by different methods' results on all mouse embryo datasets. A cell type deconvolution method cell2location is included. The two edges of box and horizontal bar inside the box represent the interquartile and median of all values, respectively.

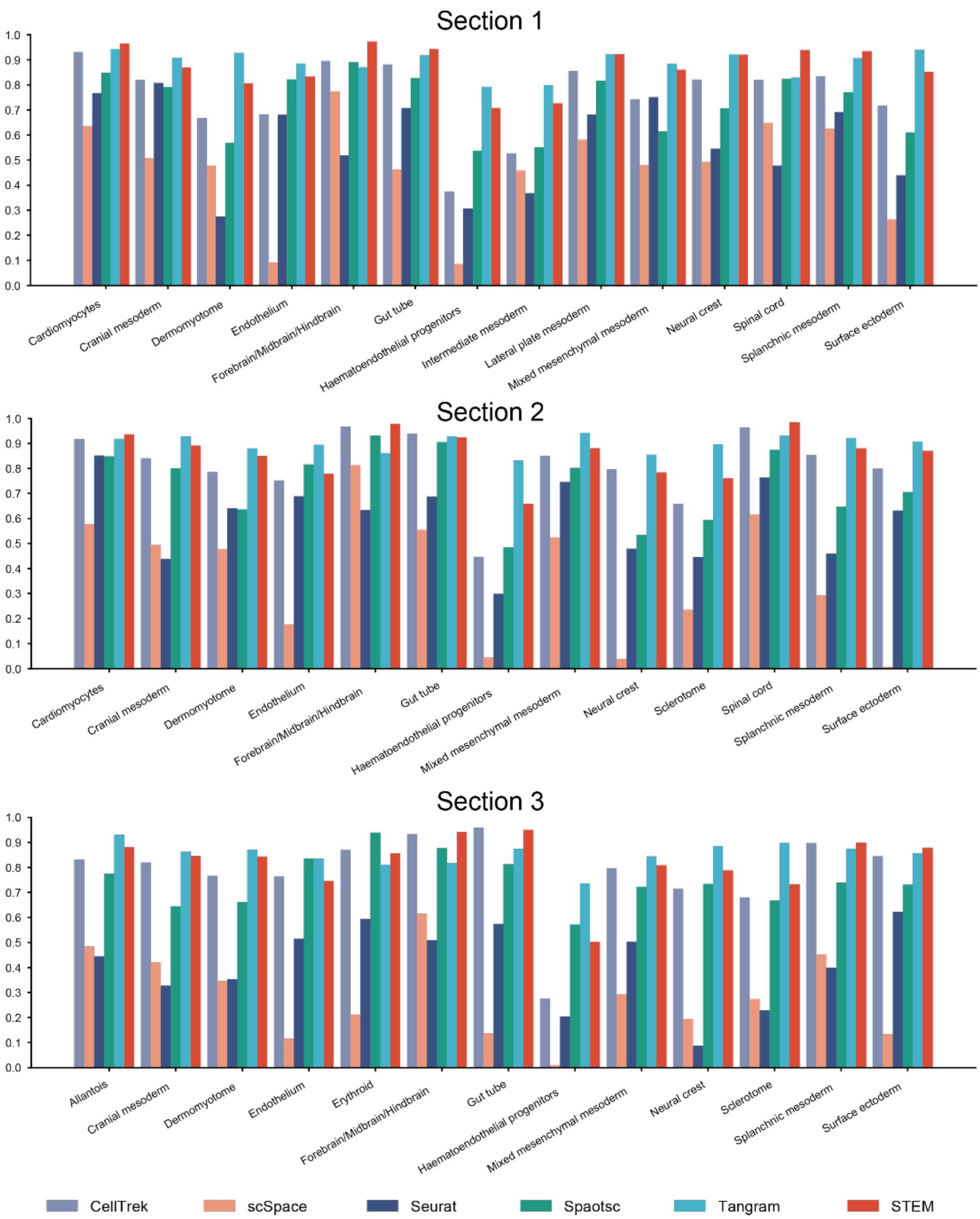

Supplementary Figure 8. The PCC of all cell types given by different methods' on all mouse embryo data.

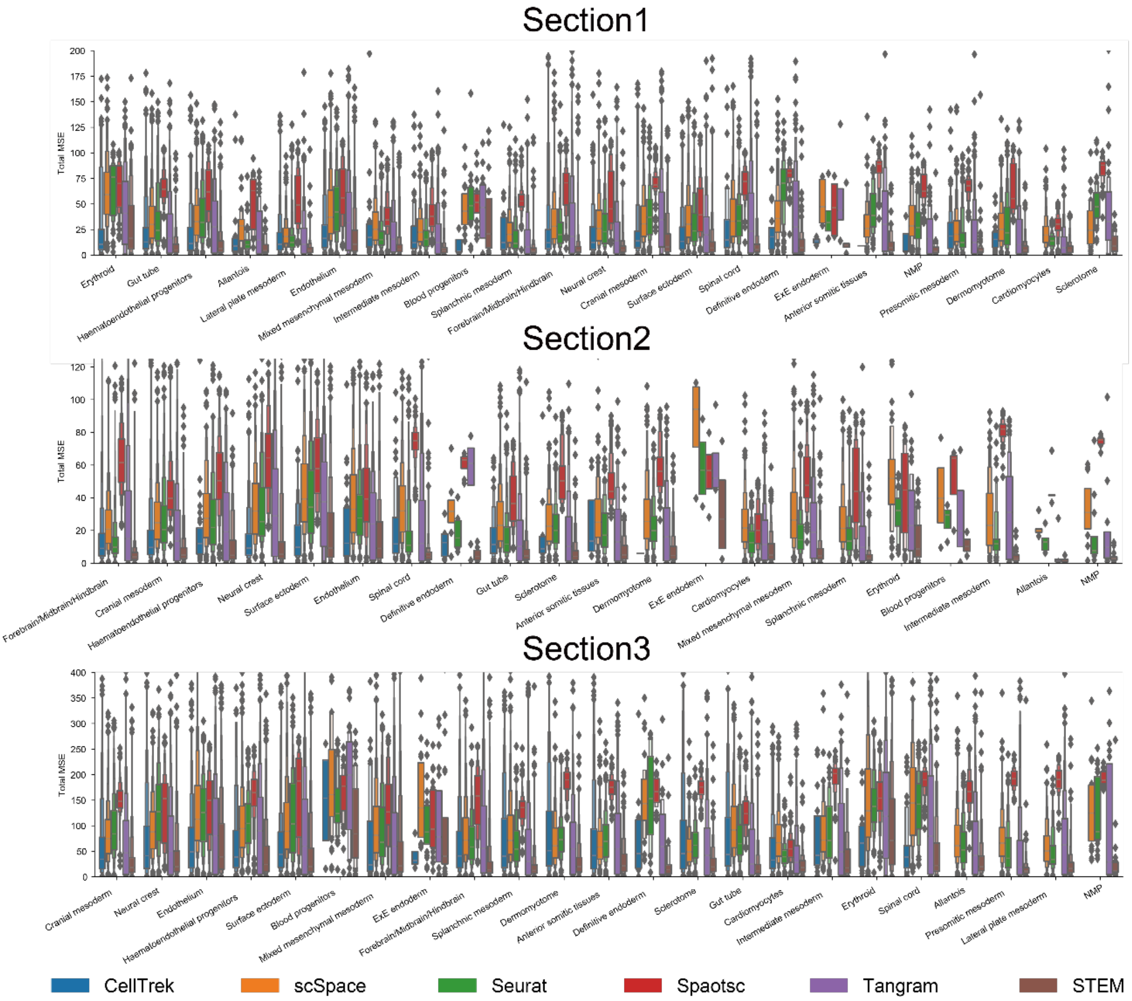

Supplementary Figure 9. The MAE of all cell types computed by different methods' on all mouse embryo data. The two edges of box and horizontal bar inside the box represent the interquartile and median of all values, respectively.

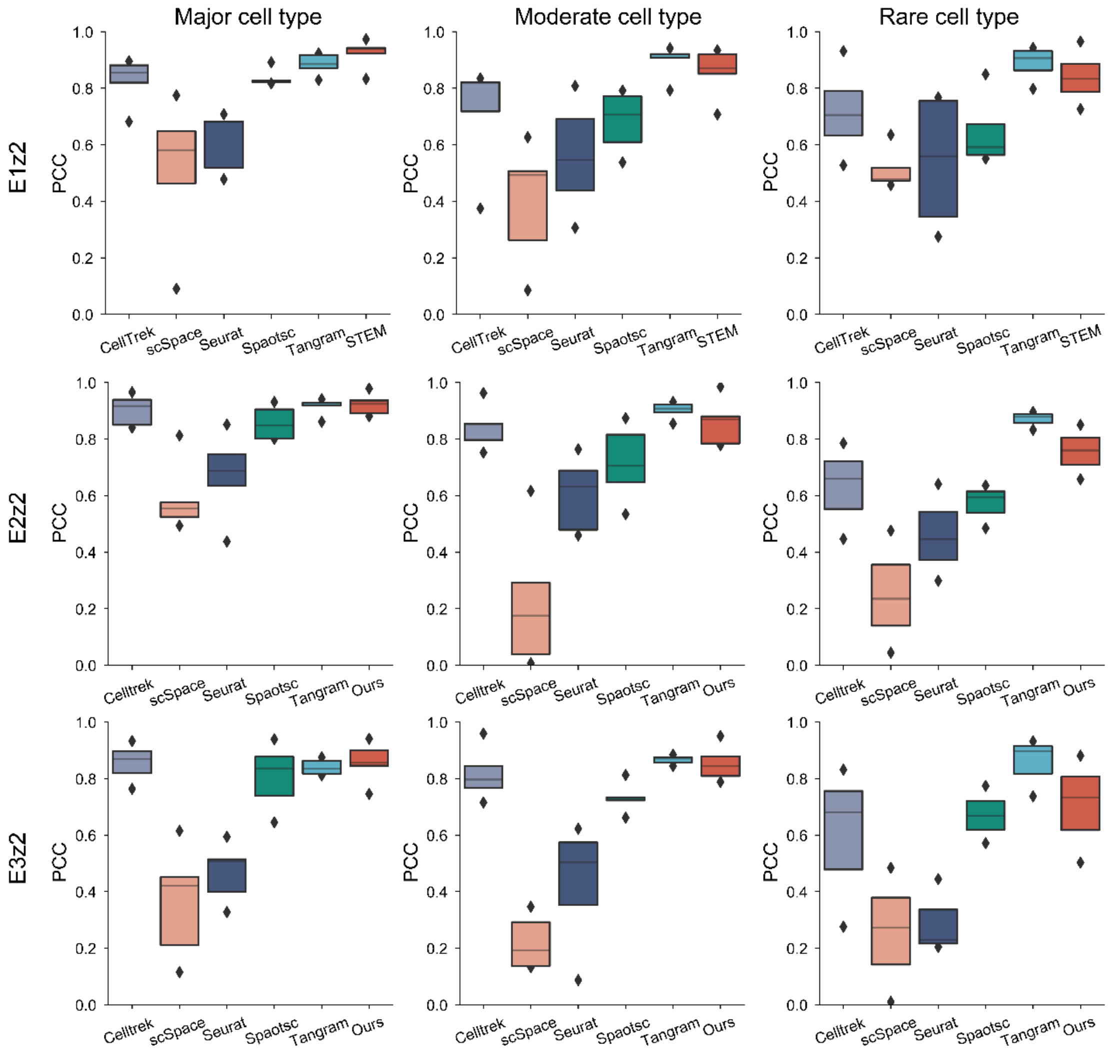

Supplementary Figure 10. The PCC results of different methods among major, moderate and rare cell types on all semi-simulated data. Each row corresponds to a dataset, and each column corresponds to one class of cell type. The two edges of box and horizontal bar inside the box represent the interquartile and median of all values, respectively.

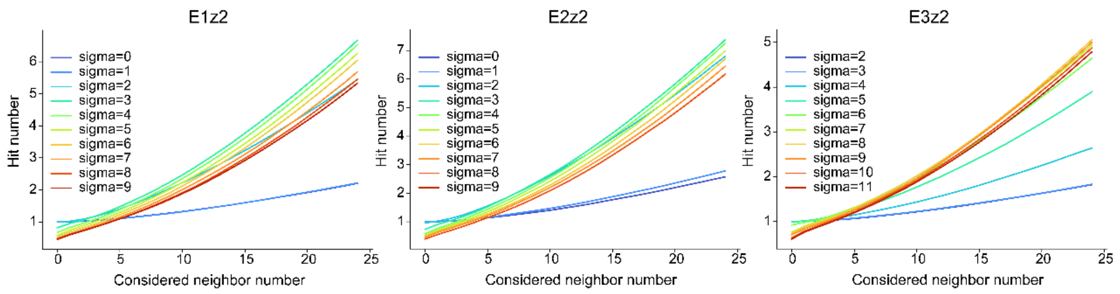

Supplementary Figure 11. The STEM hit number performance under different values of parameter σ on all three embryo data.

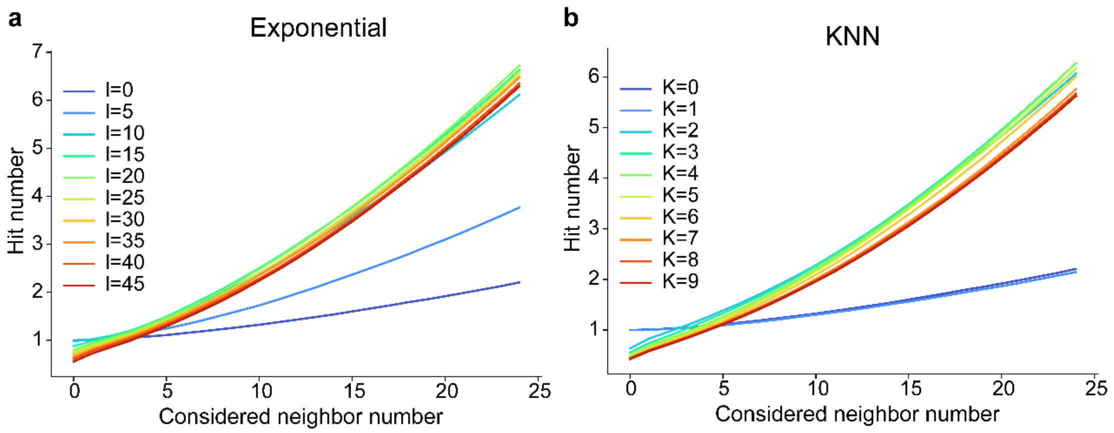

Supplementary Figure 12. The STEM hit number performance under different kernels. (a) performance under different values of parameter l in the exponential kernel. (b) performance under different values of parameter K in the KNN kernel.

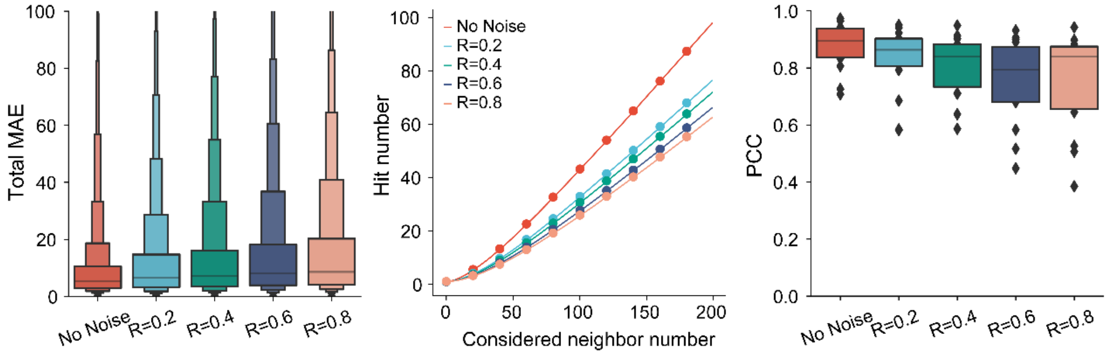

Supplementary Figure 13. The mean absolute error (MAE), hit number and Pearson correlation coefficient (PCC) performance of STEM on different noise levels (R) semi-simulated data. In PCC box plot, the two edges of box and horizontal bar inside the box represent the interquartile and median of all values, respectively. In MAE results, we used an enhanced boxplot to show more quintiles. The horizontal bar inside the box represents the median of all values. Each edge of the box represents the half percentiles of the rest data, in other words, splitting the rest data into two halves.

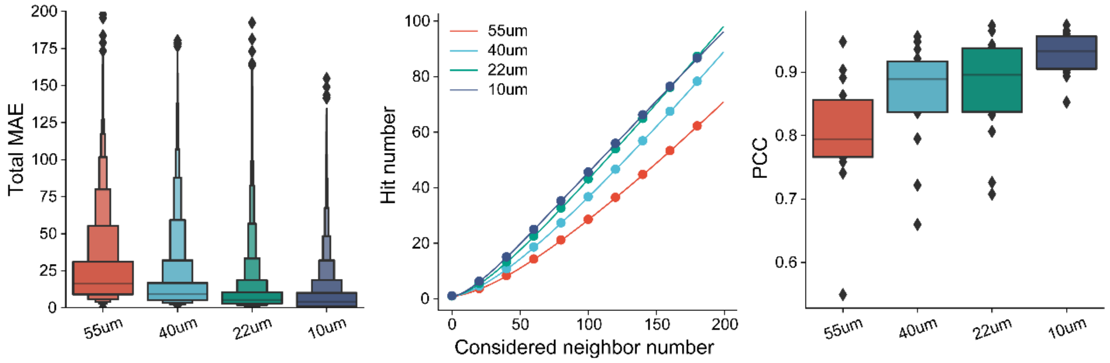

Supplementary Figure 14. The mean absolute error (MAE), hit number and Pearson correlation coefficient (PCC) performance of STEM on different resolution semi-simulated data. In PCC box plot, the two edges of box and horizontal bar inside the box represent the

interquartile and median of all values, respectively. In MAE results, we used an enhanced boxplot to show more quintiles. The horizontal bar inside the box represents the median of all values. Each edge of the box represents the half percentiles of the rest data, in other words, splitting the rest data into two halves.

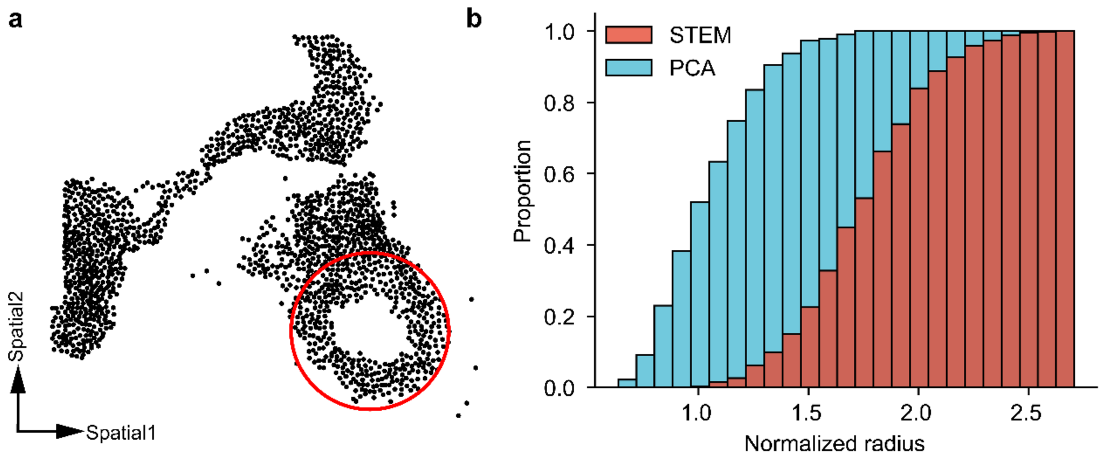

Supplementary Figure 15. The analysis of hollow structure. (a) The cells around the hollow structure (within the red circle) were selected. (b) The cumulative distribution of included cells in the sphere. Y axis: the proportion of included cells. X axis: the normalized radius.

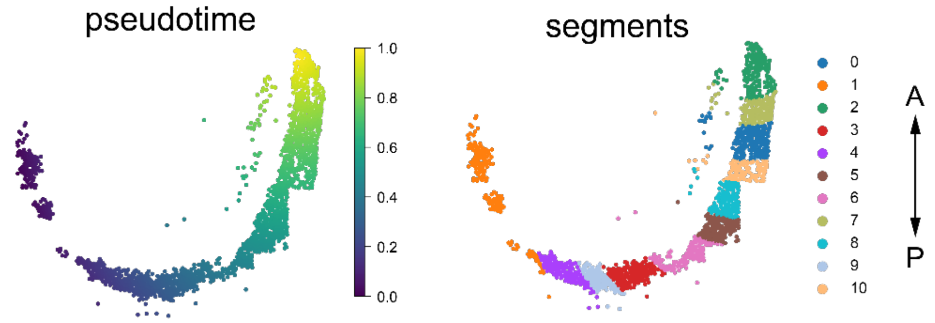

Supplementary Figure 16. The pseudo-time trajectory and segments generated in the spinal cord region. A: anterior, P: posterior.

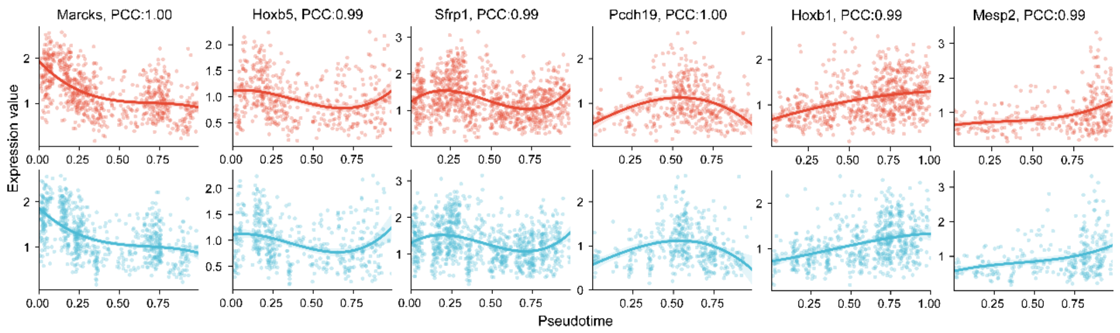

Supplementary Figure 17. Expression profiles of six marker genes along the spinal cord pseudotime. The x-axis represents the pseduotime, and the red and blue colors represent the gene's ground truth and reconstructed expression values, respectively. Each curve was obtained by fitting polynomial function of degree 3 on the corresponding expression value.

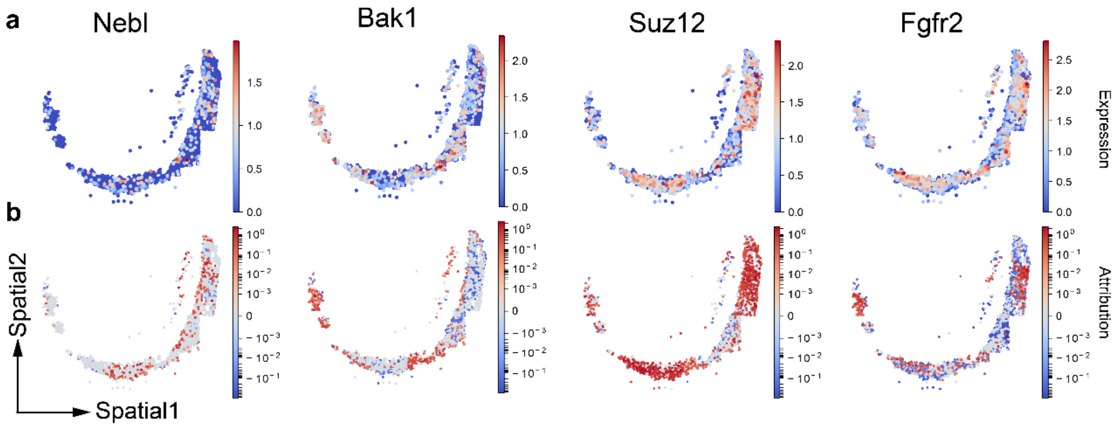

Supplementary Figure 18. Gene expression and attribution score spatial pattern in spinal cord region. (a) normalized gene expression. (b) gene attribution score. The attribution score is shown in the log form for better visualization.

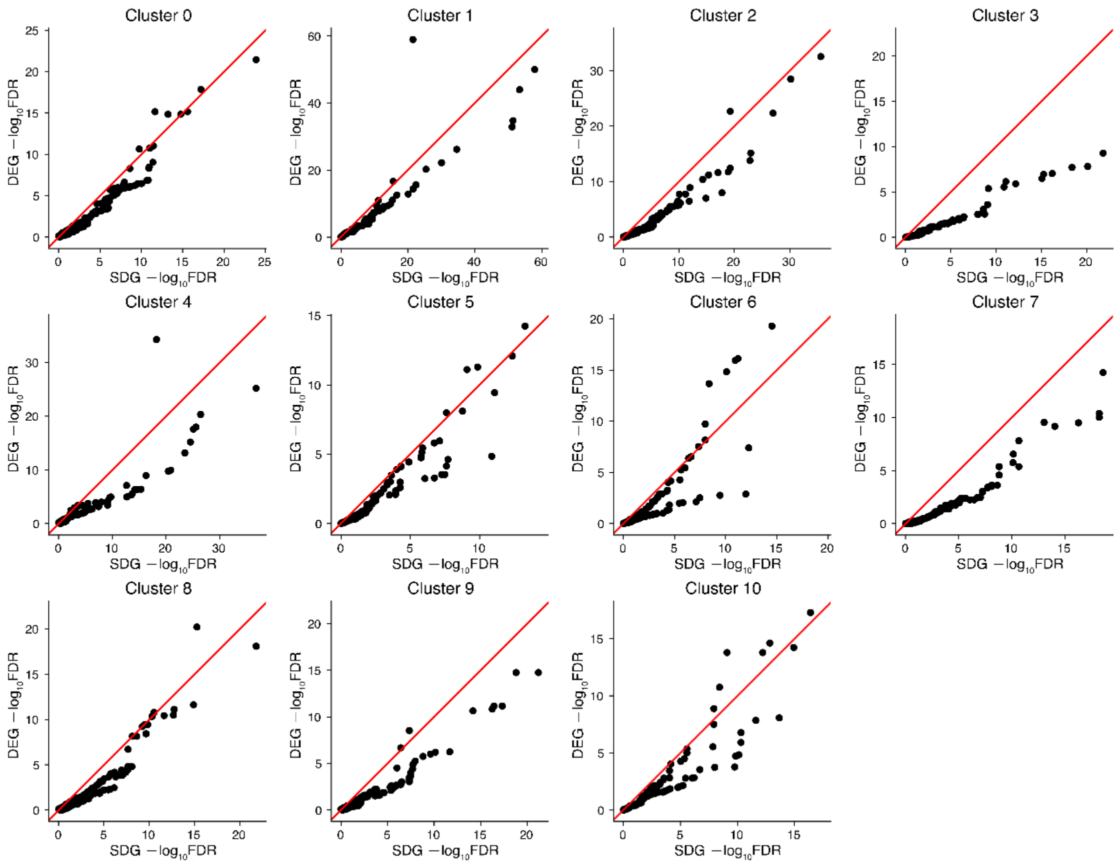

Supplementary Figure 19. The scatter plot of genes FDR values. Each subplot corresponds to a cluster in the spinal cord. Each spot is a gene, and the x and y values are the $-\log_{10}$FDR obtained from the Wilcoxon test results on attribution and gene expression profiles, corresponding to the SDG and DEG, respectively.

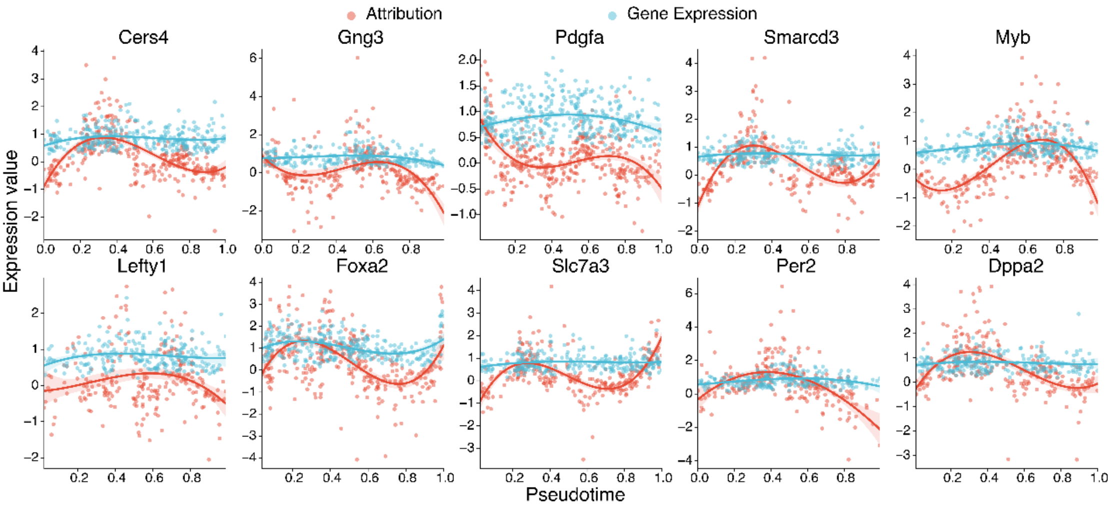

Supplementary Figure 20. Expression profiles of top 10 unique identified SDG along the spinal cord pseudotime. The x-axis represents the pseduotime, and the red and blue colors represent the gene's attribution and raw count expression values, respectively. Each curve was obtained by fitting polynomial function of degree 3 on the corresponding expression value.

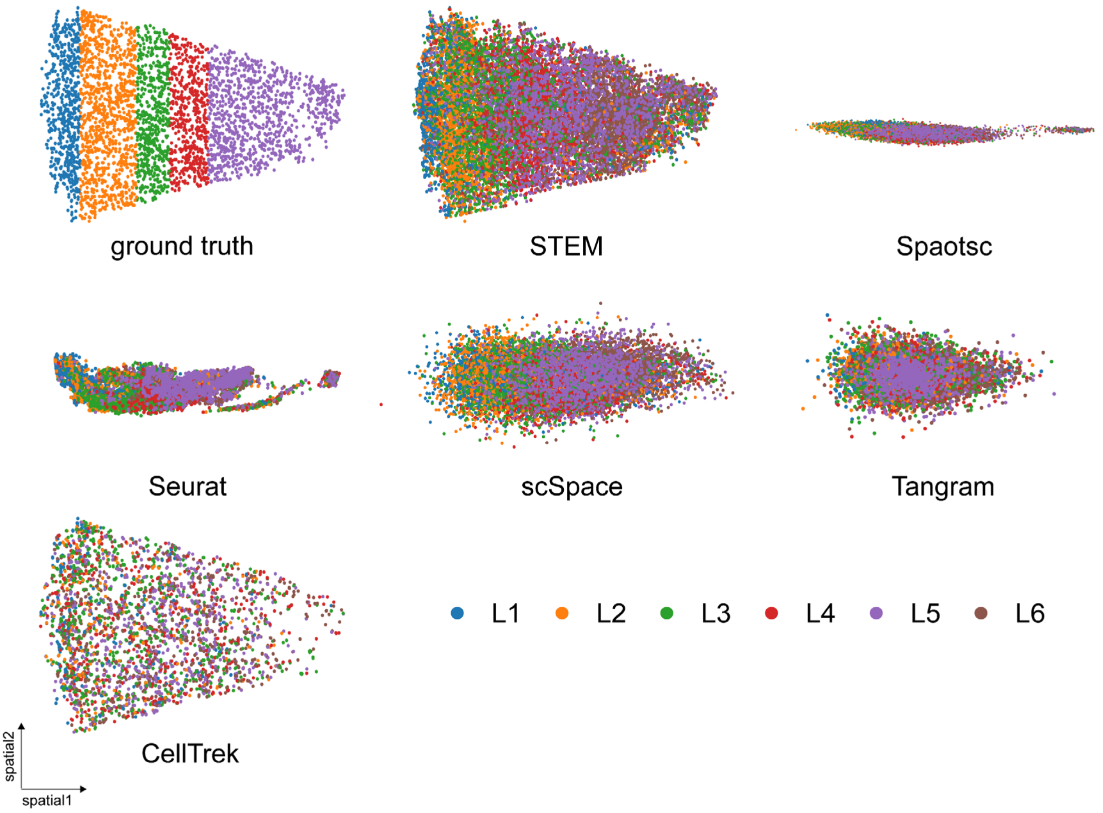

Supplementary Figure 21. The spatial reconstruction results of all methods on human MTG data.

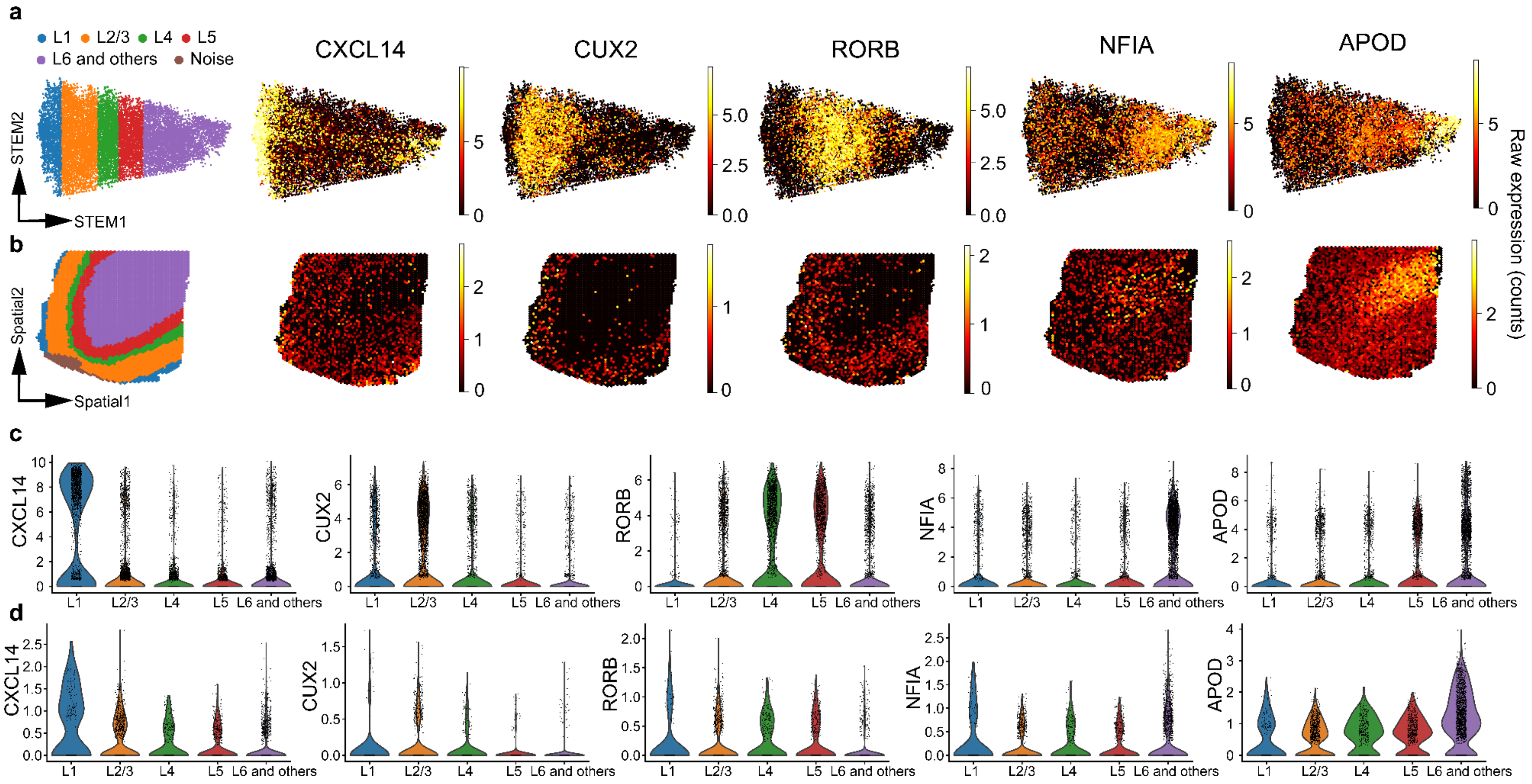

Supplementary Figure 22. Expression patterns of five region-specific genes on STEM reconstruction results and reference Visium data. (a) The region annotations and five genes' spatial expression patterns reconstructed by STEM. Raw counts values were plotted. (b) The region annotations and five genes' spatial expression patterns on the reference Visium data. (c) The violin plots of five region specific expressed genes reconstructed by STEM on human MTG data. (d) The violin plots of five region specific expressed genes on reference Visium data.

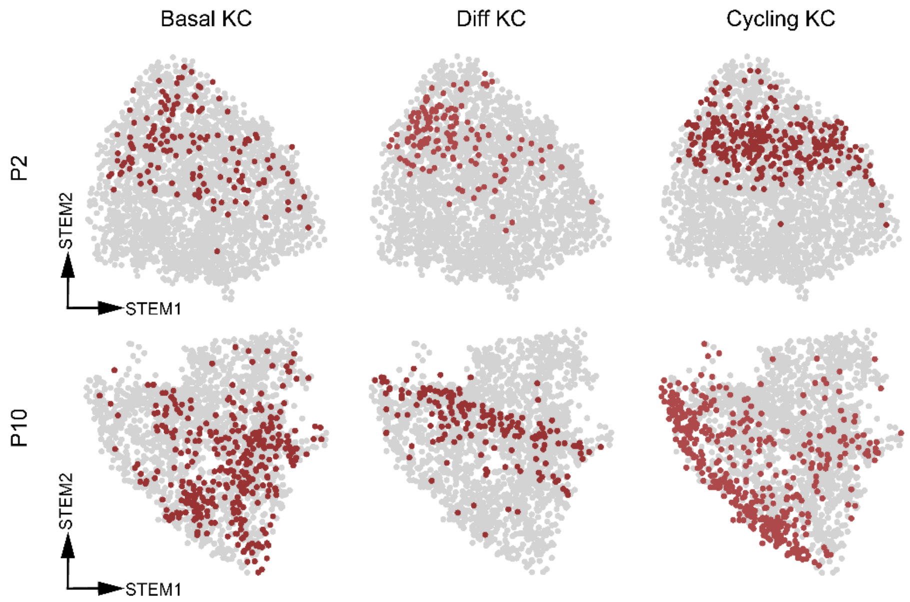

Supplementary Figure 23. Spatial distribution of different keratinocyte subtype cells on P2 and P10 slides reconstructed by STEM. Basal KC: tumor basal keratinocytes; Diff KC: tumor differentiating keratinocytes; Cycling KC: tumor cycling keratinocytes.

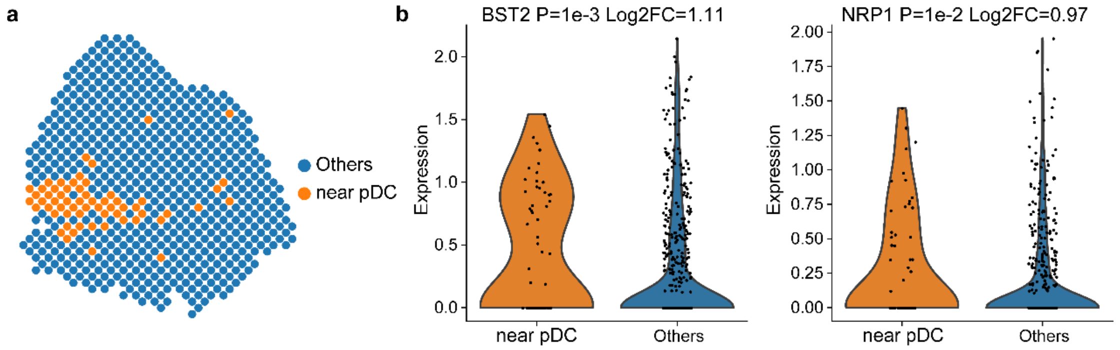

Supplementary Figure 24. Spatial and expression analysis of gene *BST2* and *NRP1* in relation to plasmacytoid dendritic cells (pDCs). (a) Spots located near the pDC single cells were in orange. (b) Violin plot of pDC enriched genes *BST2* and *NRP1*.

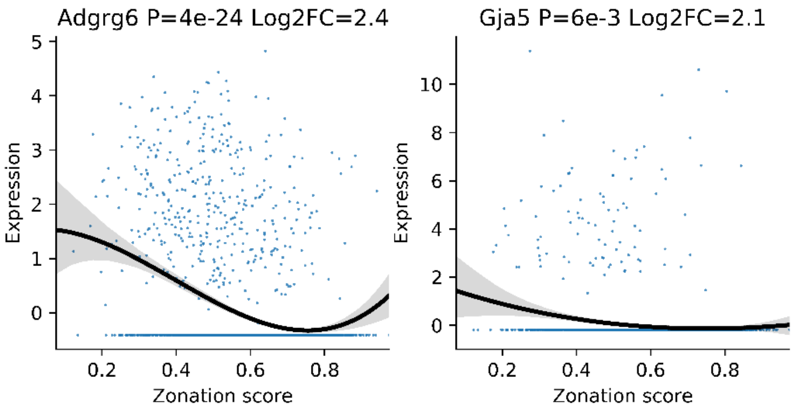

Supplementary Figure 25. Expression profiles of two endothelial marker genes along the

PV-CV axis. Each curve was obtained by fitting polynomial function of degree 3 on the corresponding expression value. The shading shows the 95 confidence interval.

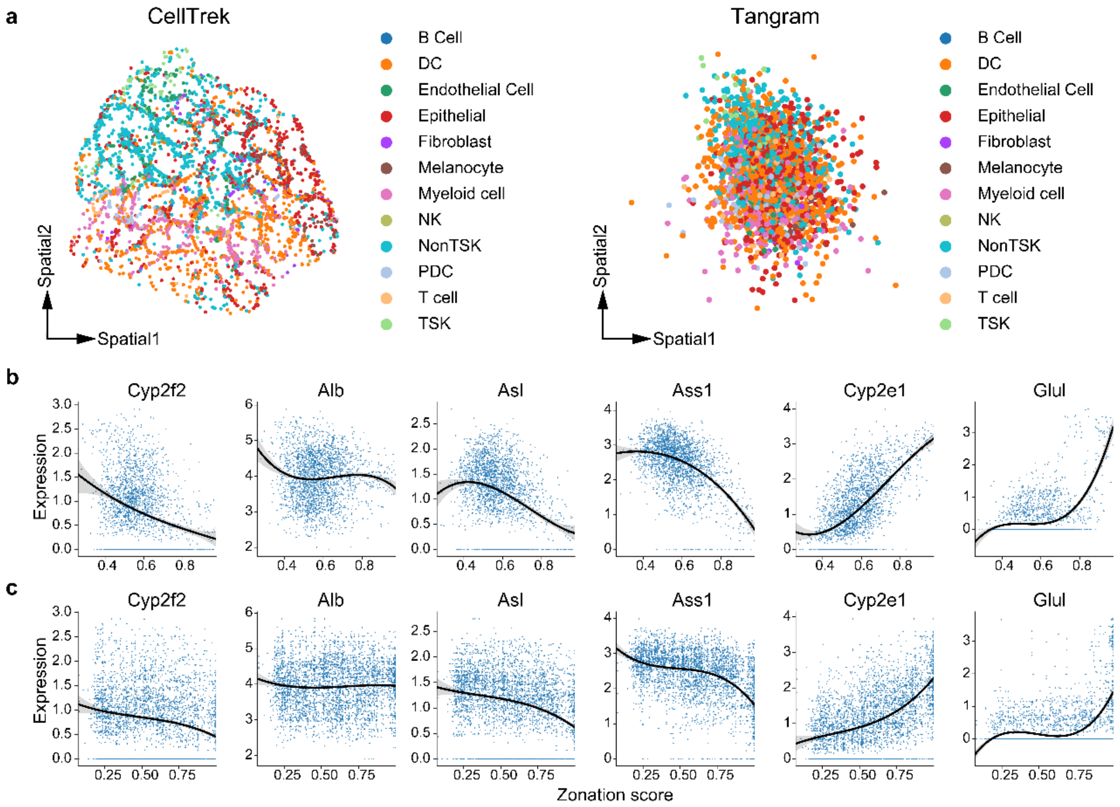

Supplementary Figure 26. CellTrek and Tangram spatial reconstruction results on two real application cases. (a) The spatial reconstructed results of CellTrek and Tangram methods on the hSCC dataset. (b) Expression profiles of six zonation landmark genes reconstructed by Tangram along the PV-CV axis. (c) Expression profiles of six zonation landmark genes reconstructed by CellTrek along the PV-CV axis. The shading shows the 95 confidence interval.

Supplementary Tables

Supplementary Table 1. The PV and CV enriched genes verified by MERFISH in previous work are also found as the DEG in the STEM results.

Gene	Annotation	Subregion	Log2FC	FDR
Aldh1b1	PV	0	2.01	1.14E-23
Cyp2f2	PV	0	0.97	9.58E-12
Etnppl	PV	0	1.06	4.27E-11
Aldh1b1	PV	1	1.66	1.41E-12
Etnppl	PV	1	1.02	2.18E-07
Etnppl	PV	2	0.87	6.66E-05
Aldh1b1	PV	2	0.87	0.022571
Cyp2e1	CV	7	0.67	1.06E-06
Cyp2e1	CV	8	1.26	8.37E-26
Gulo	CV	8	1.00	5.92E-06
Cyp2e1	CV	9	2.40	1.03E-76
Gulo	CV	9	2.49	2.62E-44
Cyp2a5	CV	9	3.87	1.29E-25
Slc1a2	CV	9	4.17	3.39E-10

Supplementary Table 2. Genes that were computationally identified as PV enriched genes by previous work are found as DEGs near PV subregions. Some genes are DEGs of more than one subregion.

Gene	Subregion	Log2FC	FDR
Sds	0	1.42	5.29E-25
Aldh1b1	0	2.01	1.14E-23
Hal	0	1.15	4.24E-19
Hsd17b13	0	1.22	4.37E-12
Cyp2f2	0	0.97	9.58E-12
Etnppl	0	1.06	4.27E-11
Hsd17b6	0	1.91	1.18E-10
Cryl1	0	1.62	7.93E-06
Sds	1	1.37	2.41E-20
Hal	1	1.04	1.59E-13
Aldh1b1	1	1.66	1.41E-12
Etnppl	1	1.02	2.18E-07
Hsd17b6	1	1.32	1.10E-03
Hsd17b13	1	0.64	4.49E-02
Sds	2	1.14	9.04E-14
Hal	2	0.91	1.37E-09
Etnppl	2	0.87	6.66E-05
Aldh1b1	2	0.87	2.26E-02
Hal	3	0.78	5.09E-06
Sds	3	0.60	8.69E-03

Supplementary Table 3. Genes that were computationally identified as CV enriched genes by previous work are found as DEGs near CV subregions.

Gene	Subregion	Log2FC	FDR
Cyp2e1	7	0.67	1.06E-06
Cyp2e1	8	1.26	8.37E-26
Cyp2c29	8	1.16	5.62E-10
Cyp1a2	8	0.99	3.00E-08
Lect2	8	1.05	5.92E-06
Gulo	8	1.00	5.92E-06
Cyp2e1	9	2.40	1.03E-76
Cyp2c29	9	2.48	2.61E-53
Cyp1a2	9	2.76	1.48E-51
Lect2	9	2.93	2.93E-45
Gulo	9	2.49	2.62E-44
Cyp2c37	9	3.34	9.35E-37
Fitm1	9	3.07	8.95E-28
Cyp2a5	9	3.87	1.29E-25
Serpina7	9	4.33	1.48E-02

Supplementary Table 4. Hit number performance under 25 considered neighbors by setting different α values

α	0	0.1	0.2	0.3	0.4	0.5	0.6	0.7	0.8	0.9	1
Hit number under 25	6.35	6.58	6.55	6.65	6.72	6.63	6.58	6.67	6.68	6.69	6.70
