# Supplementary material for: STEM enables mapping of single-cell and spatial transcriptomics data with transfer learning
Source: Commun Biol. 2024 Jan 6;7:56. doi: 10.1038/s42003-023-05640-1 (PMC10771471; doi:10.1038/s42003-023-05640-1)
Supplement: Supplementary file 3 — Description of Additional Supplementary Files [file 42003_2023_5640_MOESM3_ESM.docx]

Description of Additional Supplementary Files

**File name:** Supplementary Data 1

**Description:** The source data behind the Figure2c in the paper

**File name:** Supplementary Data 2

**Description:** The source data behind the Figure2d in the paper

**File name:** Supplementary Data 3

**Description:** The source data behind the Figure4b in the paper

**File name:** Supplementary Data 4

**Description:** The source data behind the Figure6f in the paper
